# Nonalcoholic Beverages as Sources of Nutrients in the Average Polish Diet

**DOI:** 10.3390/nu12051262

**Published:** 2020-04-29

**Authors:** Krystyna Rejman, Hanna Górska-Warsewicz, Maksymilian Czeczotko, Wacław Laskowski

**Affiliations:** Department of Food Market and Consumer Research, Institute of Human Nutrition Sciences, Warsaw University of Life Sciences, 07-787 Warsaw, Poland

**Keywords:** nonalcoholic beverages, households, energy intake, nutrient intake, free sugars

## Abstract

The aim of the study was to analyze the sources of energy, carbohydrates, 10 minerals, and 9 vitamins from nonalcoholic beverages in the average Polish diet. For the analysis, we used data from the 2016 Household Budget Survey conducted on the representative sample of the Polish population (36,886 households, *n =* 99,230). According to the source of data, we included four subgroups in analyzed food category: fruit juices, vegetable juices and mixed, mineral and spring waters, and other nonalcoholic beverages. We used the cluster analysis to assess the impact of sociodemographic and economic characteristics of the households on the structure of supplying energy and nutrients from each subgroup of the nonalcoholic beverages. Our analyses have shown that nonalcoholic beverages are primarily important in providing several nutrients: vitamin C (15.9% of the total vitamin C supply), vitamin B6 (8.9% of vitamin B supply), folates (8.5% of folate supply), carbohydrates (6.8% of carbohydrate supply), calcium (5.9% of calcium supply), and magnesium (5.5% of magnesium supply). The analysis of the consumption structure of this category of food showed that the subgroup of other nonalcoholic beverages brings more than three-fourth of carbohydrates (77%), vitamin B6 and folates (76% each), and 43% of vitamin C supplied by nonalcoholic beverages. More than half (51%) of vitamin C provided by nonalcoholic beverages comes from fruit juices and the remaining 6% comes from other juices (vegetable and mixed). In the case of minerals, mineral and spring waters consumption is important as it accounts for 65% of the calcium supply and 55% of the magnesium in nonalcoholic beverages category. The share of individual subgroups of beverages in the supply of ingredients in the diet is significantly differentiated by four socioeconomic characteristics of households: family life phase, age, socioeconomic type of household, and number of people in the household. This is particularly evident in the case of other nonalcoholic beverages, that the share of this subgroup in the energy and carbohydrates supply in the households of young people, employees (both blue-collar and white-collar workers), and families with children increases to 10%. Our results show that in order to reduce the intake of free sugars and increase the intake of deficient minerals, which is crucial in preventing noncommunicable diseases (NCDs), it is necessary to encourage consumers to replace sugar-sweetened beverages (SSBs) with water and eat fruits instead of drinking juice.

## 1. Introduction

Fluid intake in the diet is essential for life and maintaining optimal levels of hydration is important for humans to function well [1,2]. Water is involved in many body functions, being essential for digestion of food and absorption of ingredients, excretion of metabolic products and toxins, nerve transmission, and adjustment of hydroelectrolytic balance, acid–base balance, and thermal balance [2,3,4,5], and as it also acts as a lubricant and shock absorber [6]. Humans ingest water as plain drinking water, water in different beverages, water in food (inherent and added during dish preparation), and they also obtain some water from metabolism of food (350 mL/day) [3]. Approximately one-third of the daily average water intake is thought to be derived from food [7], providing on average of 500–900 mL of water [3]. Among food products, vegetables (up to 95%), fruit (up to 87%), and milk and milk beverages (87–89%) have the highest water content [8]. The remaining water requirement (even more than half) must be met by the consumption of liquids [7].

Individual body requirements for water vary according to climate and ambient temperature, physical activity, and composition of the diet [3,9,10]. Staying at high altitudes may also require a higher supply of fluids [10]. The requirement for water increases with the energy value of the diet and the consumption of protein and fiber [10,11]. Diuresis can be increased by ingestion of caffeine and alcoholic beverages [12,13,14,15].

The determination of water requirements cannot be based on a minimum intake, as it can lead to a water deficit in the body due to numerous reasons that modify requirement of water [6]. The dietary reference values for water developed as water AI (adequate intake) values in 2010 by the European Food Safety Authority (EFSA) [16] for various life-stage groups were derived from three factors: observed intakes of European population groups (water from food and beverages), desirable urine osmolality values, and desirable total water intake volumes per unit of dietary energy consumed [5]. Recommended AI for water for girls aged 14–18 years and for women is 2.0 L/day and for boys in the same age group and men equals to 2.5 L/day [17]. The US Institute of Medicine recommends higher water AI values for each population group; for adult women and men (age groups in the range from 19 years to more than 70 years of age), it is 2.7 and 3.7 L per day, respectively. Adequate Intake for total water was set to prevent deleterious, primarily acute, effects of dehydration, which include metabolic and functional abnormalities [18].

Adequate water intake is essential for maintaining health and functionality of the human body. Data from the European Hydration Research Study, covering a sample of healthy adults in three European countries (Germany, Greece, and Spain) pointed that a large number of individuals showed an inadequate hydration status on several days per week, which may have a negative health and cognitive impact on daily life [19]. In this context, it is worth noting that there are studies showing that the increase of the amount of drinking of beverages, regardless of composition and type, has not improved the hydrating status [20,21]. These results suggest that standard measures of hydration status are not sensitive enough [21].

Drinking water and other nonalcoholic beverages provides 800–1500 mL of fluid daily in the diet [3]. Over time, different ingredients have been added to improve the taste of water and to make its consumption more appealing and, more recently, beneficial to the health of the consumer [1]. The current nonalcoholic beverage market is a dynamic, growing, and highly innovative one, allowing consumers to choose according to their individual needs and preferences [22]. Soft drinks are useful to quench thirst in a pleasant way so they are the most highly consumed drinks besides water worldwide [23]. They have a very important market share with the annual growth rate of the global carbonated beverages market that reached 3.2% in the years 2014–2018 [24]. Soft drinks contain some useful compounds with antioxidant properties, but it is necessary, however, to consider some of their drawbacks related to their composition [1].

The main issue is the excessive intake of sugars from sugar-sweetened beverages (SSBs) provided little nutritional value [3,4,5]. This includes free sugars added to beverages and sugars naturally present in other beverage ingredients such as honey, syrups, fruit juices, and concentrates [6]. In recent years, it is found that excessive consumption of nonalcoholic beverages high in sugar (soft drinks, flavored water, sweetened ice-tea, etc.) can contribute to obesity [25] and a range of other preventable noncommunicable diseases such as type 2 diabetes and dental decay [26,27,28]. They have serious impact on human health, affecting many body systems such as gastrointestinal, circulatory, central nervous, and even reproductive system [23]. Drinking fruit and mixed juices is also considered in terms of sugar content [29]. Studies suggest for regular consumption of pure (100%) fruit juice (without added ingredients, e.g., sweeteners) in order to get major health-promoting compounds, which can have beneficial effects on health [30,31,32]. At the same time, lifestyle changes and increased awareness among health-conscious consumers have driven the beverage industry to develop and introduce functional drinks with added nutritional values and health-promoting benefits [33].

Water is highly variable in its mineral contents depending on the water spring, while some contribute appreciable amounts of certain minerals due to natural conditions, but most provide lesser amounts of essential minerals. Many people consume mineral waters because of the perception that it may be more healthy than other nonalcoholic beverages [7]. The nutrients found in water at potentially significant levels of particular interest are: calcium, magnesium, fluoride, sodium, copper, selenium, and potassium [7]. In the body, the electrolytes are affected by fluid balance in particular within the extracellular fluid, where the major cation is sodium and the major anion is chloride. The major cation in the intracellular fluid is potassium [34,35]. To maintain the body’s water–electrolyte balance, the excretion of water and electrolytes must be balanced by their adequate supply [18,36].

Given the importance of water and other nonalcoholic beverages in the diet, the analysis of the sources of energy and nutrients in this group of foods is important to ensure the nutritional quality of the diet. Therefore, the aim of our study was to analyze the food sources of energy and nutrients from the nonalcoholic beverage category in the average Polish diet. We also analyzed the impact of sociodemographic and economic factors on the volume of nonalcoholic beverages consumption as well as energy and nutrient supply from this food category and its subgroups.

This paper is another result of our work based on the same methodology and concerning the sources of energy and nutrients in the average Polish diet. So far, we have analyzed the food sources of protein and amino acids [37] and potassium [38] in the average Polish diet as well as the role of four food categories in energy and nutrients supplied to the diet (meat, seafood, and their products [39], milk and dairy products [40], cereal products [41], and fats and oils [42]).

## 2. Methods

### 2.1. Study Overview

In this study, we analyzed the sources of energy and nutrients from nonalcoholic beverages in the average Polish diet, based on the data from the 2016 Household Budget Survey (HBS). We took into consideration the consumption of total category of nonalcoholic beverages and of four subgroups of them (shown in the next section). The list of nutrients included carbohydrates, 10 minerals (calcium, phosphorus, sodium, potassium, iron, magnesium, manganese, iodine, copper, and zinc), and 9 vitamins (vitamin A, vitamin E, thiamin, riboflavin, niacin, vitamin B6, folate, vitamin B12, and vitamin C). We also analyzed the relationship between the energy and nutrients supplied by the beverages and the sociodemographic and economic characteristics of households participating in the Central Statistical Office (CSO) survey. HBSs are carried out by CSO in Poland, by Social Surveys and Living Conditions Statistics Department. The surveys are based on the representative method of sample selection which allows for the generalization of the results to the whole population of households. In 2016, the number of surveyed households was 36,886.

### 2.2. Sample Selection Method

Each household participating in the survey kept a special diary for a month, recording their expenditures, quantitative consumption, and incomes. In order to draw the sample, a two-stage, layered drawing scheme was used, with different selection probabilities in the first stage. The primary sampling units were statistical regions covering the whole territory of Poland and those for the second stage are dwellings. In 2016, 3132 dwellings were surveyed every month (this could have allowed to gather the sample of households inhabiting 37,584 dwellings during this year). Ultimately, the number surveyed was 36,886 households, totaling 99,230 people [43]. In our analysis, to calculate the volume of food consumption per person per month, we used the data obtained by CSO from this number of households.

The monthly rotation of households assumes that in every month of the year, a different group of households participates in the survey. Food consumption reported by households comprises three sources of food procurement: purchases of food, self-supply (products taken from the farm, plot, or own food business), and food received free of charge, e.g., as a gift, from the family, from other people with surplus food, etc. Food which has entered the household is considered as food which has been consumed in the household. So, it is clear that data on quantitative food consumption in CSO surveyed households do not cover food consumed out-of-home, e.g., in food service establishments [43].

### 2.3. Data Collection

Data on household food consumption from the HBS cover 91 subgroups of food. In our analysis, we combined them into 13 main categories, i.e., meat and meat products; cereal and grain products; milk and dairy products; sugar and sweets; snacks; vegetables and vegetable products; fruits and fruit products; eggs; seafood; coffee, tea, and cacao; nonalcoholic beverages; alcoholic beverages; and fats and oils. A detailed classification has been published in our previous publications [37,39,41] based on the food classification scheme published in the literature [44,45,46], applied by FAO [47], and food products commonly consumed by Polish consumers [43,48].

In this paper, we present an analysis of nonalcoholic beverages as a source of energy and nutrients in the average Polish diet. The group of nonalcoholic beverages in the HBS includes four subgroups:

1. fruit juices, including fruit drinks and nectars,
2. vegetable juices and mixed (fruit–vegetable),
3. mineral and spring water (bottled),
4. other nonalcoholic beverages, including sweetened and unsweetened, carbonated and noncarbonated beverages, flavored (and sweetened) mineral waters (which together form a group called soft drinks), energy and sport drinks, “vegetable drinks”/milk substitutes (e.g., drinks from soya, oat, almonds, rice, etc.), and cordials and concentrates for the preparation of beverages.

The category of nonalcoholic beverages does not contain tap water consumption as well as milk drinks (yoghurt, buttermilk, kefir, whey drinks, flavored milk, etc.), as they are included in the category of milk and dairy products. This group also does not include home-drinking coffee and tea, as the products for their preparation are classified in a separate category.

### 2.4. Data Analysis

The course of our calculations and analyses included:

1. data collection on quantity of the monthly consumption of 91 food subgroups (in grams, kilograms, liters) per individual household (from Central Statistical Office database);
2. conversion of consumption quantity into one person per month in each household (in grams, kilograms, liters);
3. conversion of consumption data into energy and nutrients content (in kcal, g, mg, µg per day) in 91 subgroups of consumed food products in each household;
4. calculation of the average energy and nutrients supply in each food subgroup (in kcal, g, mg, µg per day per person) in the sample of all households;
5. calculation of the average energy and nutrients contribution from each food subgroup to the average Polish diet (in %);
6. analysis of impact of sociodemographic and economic variables of households on the level and structure of energy and nutrients’ supply from nonalcoholic beverages.

The quantitative data on the consumption of nonalcoholic beverages were converted into energy and nutrients they provide, using the most recent official version of the “Nutritive Value Tables for Foods and Meals” (4th ed.) in Poland [8] and the R program v3.0.2 (Copyright © 2018, The R Foundation for Statistical Computing, Vienna, Austria), constituting the appropriate environment for statistical calculations [49,50,51]. We then calculated the average energy supply and the average supply of individual nutrients from nonalcoholic beverages, which were expressed in kcal, g, mg, µg per person per day. This allowed us to determine the average energy and nutrient contribution (in %) to the average Polish diet from each subgroup.

To analyze the impact of sociodemographic and economic factors on the volume and structure of nonalcoholic beverages consumption, we conducted cluster analysis [52,53,54] using the Neural Networks module in the Statistica 13.3 (Copyright 1984-2917, TIBCO Software Inc., Palo Alto, CA, USA) and Kohonen Neural Network [55]. We identified 3 clusters based on 14 variables characterizing the sample of surveyed households: education, income level (quintile group), degree of urbanization of the place of residence, socioeconomic type of household, size of the village, usage of agricultural land, self-assessment of financial situation, number of people in a household, region, family life phase, self-assessment of nutrition in a household, age and sex of the head of household, and month of participation in the survey. For each of these characteristics, we calculated the Cramer correlation to indicate those that most strongly determine the consumption of nonalcoholic beverages. 

## 3. Results

### 3.1. Nonalcoholic Beverages as Sources of Energy and Nutrients

Our analysis concerns the supply of energy, carbohydrates, 10 minerals, and 9 vitamins from nonalcoholic beverages in the average Polish diet (Table 1).

The whole category of nonalcoholic beverages provides 3.3% of the total energy in the average Polish diet (amounting to 2261 kcal/person/day). Energy from this category comes exclusively from carbohydrates, and nonalcoholic beverages in the average Polish diet provide 6.8% of total carbohydrates (carbohydrate intake in the average Polish diet is 225–300 g/person/day). More than three-fourth of this energy and carbohydrates consumption comes from the “other nonalcoholic beverages” subgroup and less than one-fifth from the “fruit juices” subgroup.

Calcium is the highest among the minerals supplied by nonalcoholic beverages in the average Polish diet. The second highest mineral is magnesium, but the share of each mineral is less than 6%. They are derived primarily from mineral and spring water (3.9% and 3.0%, respectively), followed by the subgroup of other nonalcoholic beverages (1.2% and 1.3%, respectively). The share of nonalcoholic beverages in the supply of potassium (4.5%) and copper (3.5%) can also be indicated. About half of these minerals are mainly derived from the subgroup of other beverages. The share of nonalcoholic beverages category in the supply of other minerals is very small, with 2.6% for iron and manganese and even less for the others, from 1.58% for sodium to 1.03% for phosphorus and zinc. Sodium, like calcium and magnesium, comes mostly (more than 80%) from mineral and spring water. Subgroup of other nonalcoholic beverages is first in the supply of the remaining seven minerals, so neither fruit juices nor vegetable juices and mixed are first only in the supply of iodine; fruit juices are not first in providing any of the minerals.

However, the results of the analysis showed the importance of fruit juices as the first source among subgroups of nonalcoholic beverages of four vitamins. The whole category of nonalcoholic beverages provides 15.9% of vitamin C in the average Polish diet and slightly more than half of this amount (8.1%) comes from fruit juices. The subgroup “other beverages” comes second in the supply of vitamin C (6.8%). The category of nonalcoholic beverages also provides almost 9% of vitamin B6 as well as folate in the average Polish diet, with a subgroup of other beverages having the largest share (6.8% and 6.5%, respectively). Fruit juices come first in the supply of thiamine (1.1%), riboflavin (0.6%), and niacin (0.6%), while vegetable juices and mixed is the subgroup suppling the most of vitamin A (2.3%). The category of nonalcoholic beverages provides 3.7% of vitamin A in the average Polish diet.

### 3.2. Impact of Sociodemographic and Economic Factors on the Level and Structure of Nonalcoholic Beverages Consumption

In order to analyze the impact of sociodemographic and economic characteristics of households on the consumption of the discussed subgroups of nonalcoholic beverages, three clusters were distinguished. Four factors determined the consumption of these products and the supply of energy and nutrients to the greatest extent: family life phase, age, socioeconomic type of households, and number of people in the household (Table 2).

The factors describing the most numerous groups of households were presented as cluster 1, constituting more than half of the surveyed sample (51.3%). These are older people, and older marriages, which are retired (28%) or still working (26%), mostly over 50 years of age (71%), pensioners (40%), and employees (20% in worker positions and 19% in nonworker positions), living in a 2-person (39%) and 1-person households (26%). Cluster 2, including one-fifth of the surveyed population, with approximately equal share of six groups of households distinguished in terms of family life cycle phase, mainly those of three age groups between 30 and 70 years of age (almost 80% in total), employees (26–27% of the two types each), and living in 2-person (30%) and 3-person (23%) households. Cluster 3, constituting 30% of the surveyed households, can be described as the group of young people (40% of people under 40 years of age), families with children at preschool and school age (47% in total), economically active (the highest share of blue-collar workers and white-collar workers: 63% and self-employed: 9%), and living in 2–4—person households (approximately equal share of each group of 24%) (Table 3).

**Figure 1:**
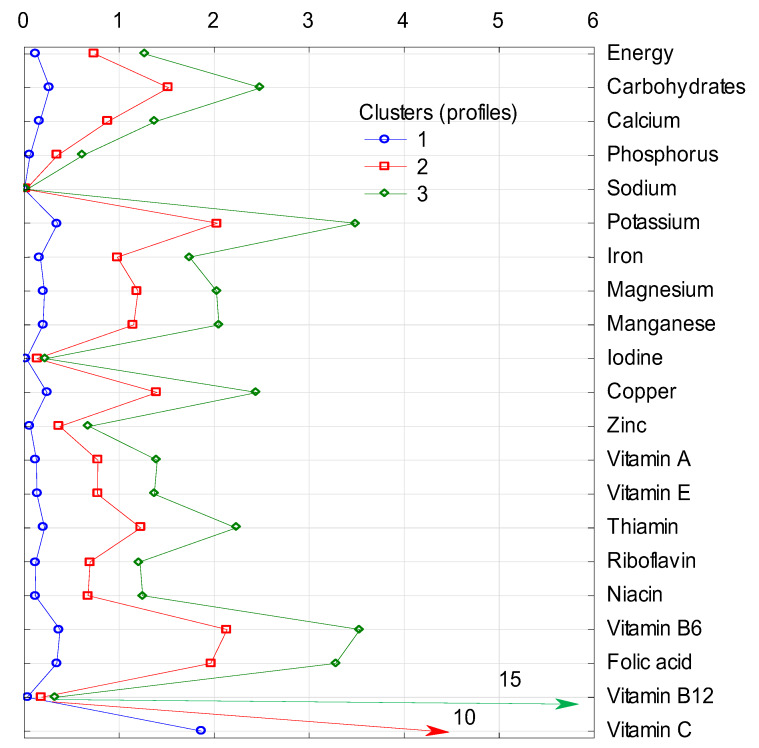
Cluster analysis: supply of energy and analyzed nutrients (in %) from the subgroup of fruit juices to diets in individual clusters. 1, 2, 3—number of clusters; characteristics of clusters are presented in Table 3.

**Figure 2:**
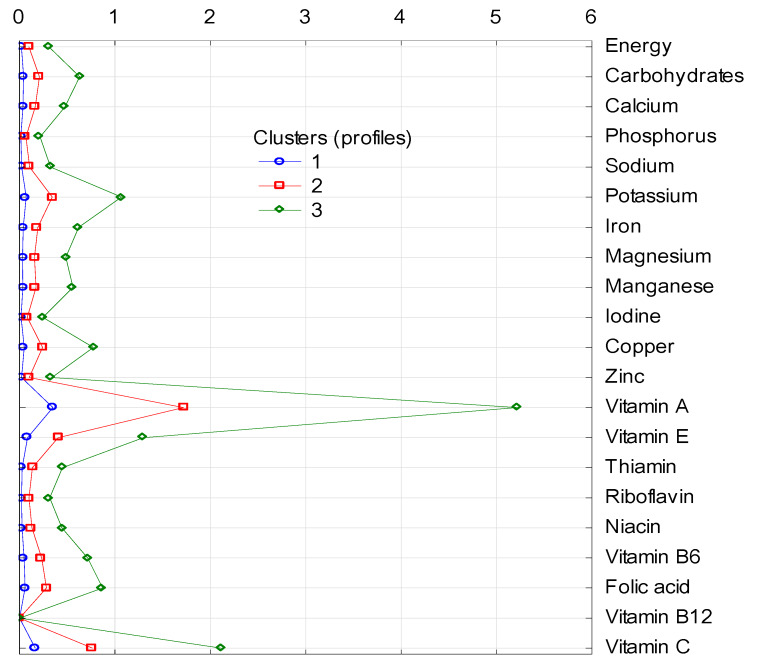
Cluster analysis: supply of energy and analyzed nutrients (in %) from the subgroup of vegetable juices and mixed to diets in individual clusters. 1, 2, 3—number of clusters; characteristics of clusters are presented in Table 3.

**Figure 3:**
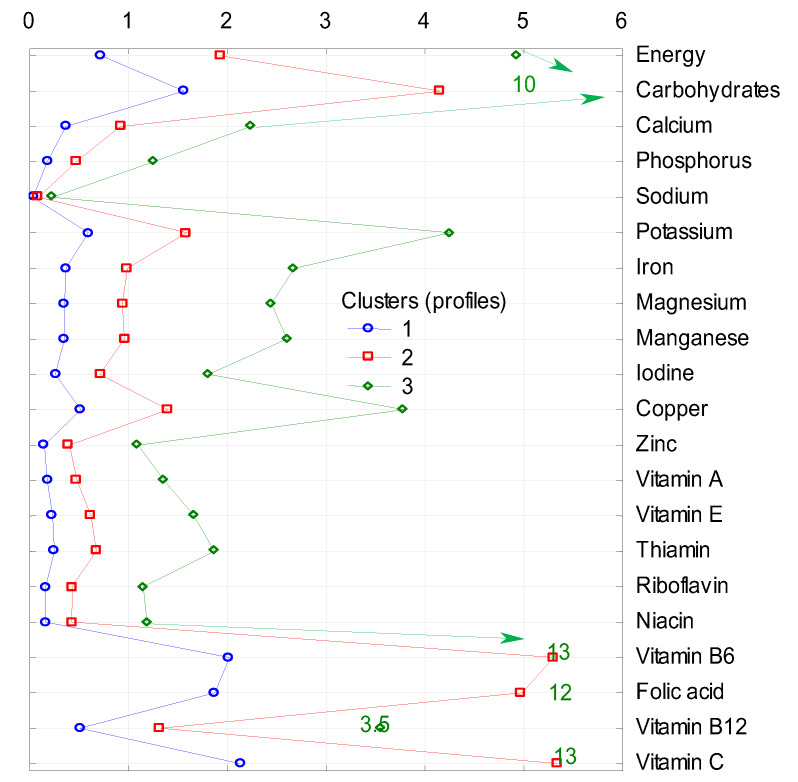
Cluster analysis: supply of energy and analyzed nutrients (in %) from the subgroup of other nonalcoholic beverages to diets in individual clusters. 1, 2, 3—number of clusters; characteristics of clusters are presented in Table 3.

**Figure 4:**
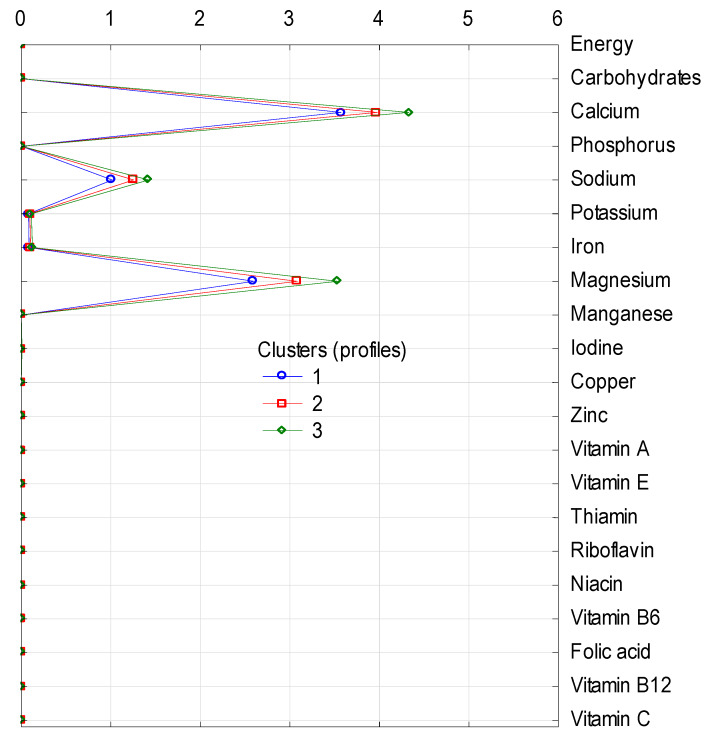
Cluster analysis: supply of energy and nutrients (in %) from the subgroup of mineral and spring water to diets in individual clusters (the graph shows only the nutrients present in waters). 1, 2, 3—number of clusters; characteristics of clusters are presented in Table 3.

The clusters differ in terms of the share (in %) of energy and analyzed nutrients in the average diet, as cluster 1 is the group with the lowest share and cluster 3 with the highest share in energy and all nutrients supply in each of four groups of nonalcoholic beverages included in the analysis (Figure 1, Figure 2, Figure 3 and Figure 4).

In case of fruit juices as a source of energy and nutrients supplied in the diet, a large variation between clusters was found (Figure 1). Fruit juices in cluster 1 provided less than 0.5% of energy and all nutrients included, except for vitamin C, for which they provided almost 2% of this vitamin. In cluster 2 and 3, fruit juices provided 10% and 15% of vitamin C, respectively. In both clusters, they contributed more than 2% of potassium and vitamin B6, and in cluster 3, also the contribution was over 2% for carbohydrates, magnesium, manganese, copper, thiamin, and folates (Figure 1).

Vegetable juices and mixed had a much smaller role, as their share in the supply of energy and most of the nutrients was less than 1% (Figure 2). In cluster 2, the only exception was almost 2% of vitamin A supply, and in cluster 3, the exceptions were potassium (just over 1%), vitamin A (over 5%), vitamin E (over 1%), and vitamin C (just over 2%).

The share of the subgroup of other nonalcoholic beverages (Figure 3) in the supply of energy and nutrients in the diet in individual clusters was the most varied. In cluster 1, as in the case of fruit juices, they provided less than 0.5% of most of the nutrients included, exceptions were energy (less than 1%), carbohydrates (over 1.5%), vitamin B6, folates, and vitamin C (around 2% share). In cluster 3, the subgroup of other nonalcoholic beverages provided 10% of energy and carbohydrates (in cluster 2, much less, about 2% and 4%, respectively), more than 2% of phosphorus, iron, magnesium, and manganese, more than 4% of potassium, and less than 4% of copper and vitamin B12. The role of this subgroup of beverages was clearly the biggest in the case of vitamins B6 and C, because their share in cluster 3 was 13% (in cluster 2, it was over 5% for each of these two vitamins), and in the case of folates, it was 12% (in cluster 2–5%).

In turn, minimal differences between clusters were found in the case of mineral and spring waters as a source of nutrients (Figure 4). This group provided calcium, magnesium, and sodium and their share was 4%, 3%, and 1%, respectively. Moreover, waters had a marginal share in the supply of potassium and iron.

## 4. Discussion

The purpose of our study was to determine the supply of energy and nutrients from nonalcoholic beverages in the average Polish diet, which were divided into four subgroups in this food category: fruit juices, vegetable juices and mixed, mineral and spring waters, and other nonalcoholic beverages. We compared our results with those from other countries, including the United States [46], Belgium [56], New Zealand [57], Australia [58], and France [59].

Our research has shown that nonalcoholic beverages provide 6.8% of total carbohydrate supply, which is twice the share of this category in the energy supply (3.3%). The subgroup of other nonalcoholic beverages, which dominates the supply of energy and carbohydrates from the discussed food category, provides 2.5% and 5.3% of carbohydrates in the average Polish diet. This subgroup includes soft drinks that are popular, everywhere and easily accessible (sweetened and unsweetened, carbonated and noncarbonated beverages, and flavored and sweetened mineral waters), energy and sport drinks, “vegetable drinks”/milk substitutes, and cordials and concentrates for the preparation of beverages [48].

In the average American diet, compared to the Polish one, the share of soft drinks in carbohydrate and energy supply was substantially higher (which is not surprising given the habit of high consumption of soft drinks) and amounted to 11.4% and 5.4%, respectively [46]. In the average Australian diet, the share of nonalcoholic beverages in the energy supply was lower than in the American diet, but still higher than in the Polish diet. This food category provided 5.2–5.7% of energy (depending on gender), including fruit and vegetable juices and drinks, which had a higher share (2.1–2.4%) than in the Polish diet, while soft drinks, flavored mineral waters and electrolyte drinks had a similar share (2.0–3.0%) as a subgroup of other nonalcoholic beverages in the Polish diet.

The supply of carbohydrates from nonalcoholic beverages in the average Australian diet was 1.4–1.7 times higher than in the Polish diet and amounted to 9.6–11.7%, including fruit and vegetable juices and drinks (4.5–4.8%) and soft drinks, flavored mineral waters, and electrolyte drinks (4.3–6.9%).

Additionally, data from Australian survey show that the intake of sugar from nonalcoholic beverages in the average Australian diet was 20.4–26.0%, divided into fruit and vegetable juices and drinks (10.1–10.4%) and soft drinks, flavored mineral waters, and electrolyte drinks (9.3–15.5%) [58].

Data on the average New Zealand diet show that compared to the average Polish diet, nonalcoholic beverages provide half as much energy (the share of this category is 5%) and a quarter as much carbohydrates (8.6%). Carbohydrates in nonalcoholic beverages are mainly sugars, and therefore, this category provides 16.7% of sugars in the New Zealand diet. However, it should be noted that in the quoted survey, all tea, coffee and substitutes, hot chocolate drinks, juices, cordials, soft drinks, water, powdered drinks, and sports and energy drinks were considered as nonalcoholic beverages [57].

In the average Belgian diet, nonalcoholic beverages provided 25% of mono/disaccharides, including carbonated/soft/isotonic drinks (−18.5%) and fruit and vegetable juices (6.2%) [56]. In the diet of adults in the UK, nonalcoholic beverages provide 21% free sugars, making them a third source of these unnecessary food ingredients. They have exactly the same proportion in the diet of children aged 1.5–3 years and 1 percentage point higher among children aged 4–10 years, making them a second source of free sugars. The worst situation in terms of risk of diet-related diseases occurs among teenagers aged 11–18 years as nonalcoholic beverages are the main source of free sugars in their diet (33%). “Soft drinks, not low calorie” provide 7%, 10%, and 22% of free sugars in these age groups of children, respectively [60]. For the adult English population, a smaller share of soft drinks in sugar supply was obtained (21%), but for children aged 11–18 years nonalcoholic beverages provide the main source of free sugars (33% of total daily supply). Within the nonalcoholic beverages group fruit juices contributed 10% to free sugar intake [31]. Representative surveys in Belgium, France, Denmark, Hungary, Ireland, Italy, Norway, The Netherlands, Spain, and the UK show that nonalcoholic beverages have contributed more to total sugars intakes in children than in adults [61]. Data on the consumption of added sugars by children are availabel for three countries: France, The Netherlands, and the UK. The data show that the share of beverages in the supply of these ingredients is much higher in France and in The Netherlands. In France, it is 20% and 21% for girls and boys, respectively, and in The Netherlands, it is 31% and 34%, respectively. In the diet of UK girls, beverages contribute 35% of added sugars, and in boys, it is 37%, which is 2 percentage points less than in men [61].

The results presented above allow us to conclude that in terms of carbohydrate contribution by the subgroup of other nonalcoholic beverages to the average diet, the Polish diet has performed quite favorably compared to other countries’ diets. However, the results of our analysis also showed that the share of this subgroup of beverages in households of young, working people with children (cluster 3, Figure 3) increases to 10% in the supply of energy and carbohydrates. Studies show that in Poland, older boys and young men consume sweetened carbonated beverages in the highest amounts; also, among older girls and young women consumption of sweetened carbonated beverages is relatively high. High consumption of these drinks is most often combined with the risk of obesity, due to the high content of monosaccharides. It is also likely to increase the risk of type 2 diabetes [62]. Therefore, also in Poland, attention is drawn to the need to reduce the consumption of soft drinks, especially by children and young people. In these population groups, overweight and obesity rates are alarming, and among the causes of such worrying indicators is the excessive consumption of soft drinks. In a representative sample of Polish elementary, middle school, and secondary school students obtained in the OLAF study, the overall prevalence of overweight and obesity was 16.4% (18.7% in boys and 14.3% in girls) [63]. The results of the Health Behaviour in School-aged Children (HBSC) survey show that the percentage of Polish children who think they are too fat is the highest in Europe, and the highest rate is among 11-year-old children (41% for girls and 31% for boys) [64].

Food and nutrition policy experts in the frame of initiatives regarding intake of healthy foods suggest for Poland to restrict the availability of SSBs on the premises of educational establishments and to apply a tax on SSBs to disincentive consumption, particularly among children and adolescents. The latter initiative is a robust and wide-reaching intervention (the introduction of which would require more effort or investment than the former but would most likely have a greater public health impact) [65]. In line with this, the Polish Ministry of Health has presented a bill to impose an additional fee on sweetened drinks (PLN 0.7-0.8/item), aiming to combine health and fiscal benefits. Up to now, different countries have passed legislation to introduce or increase taxes on specific food items such as soft drinks, sweets, chocolate, ice cream, or other unhealthy foods, often aiming to combine fiscal and health benefits. A specific form of taxation on SSBs and other sweetened products, a so called “sugar tax,” has been introduced in nearly 40 countries around the world. In Europe, the tax has been imposed in Great Britain, Isle of Man, Ireland, France, Hungary, and Portugal. Elsewhere in the world, among others, Peru, Chile, and Mexico have taxed sweetened drinks since 2014 [66,67,68]. In Mexico, purchases of taxed beverages decreased by 5.5% in 2014 and 9.7% in 2015, yielding an average reduction of 7.6%. Households at the lowest socioeconomic level had the largest decreases in purchases of taxed beverages in both years [69]. Although data are limited, emerging evidence indicates that food taxes can influence consumption [70]. Tackling obesity is a pressing concern for many governments, and sugar taxes often look like an appealing solution. World Health Organization (WHO) is among proponents, taking the view that sugary drinks are a major source of sugar in the diet, especially amongst children and adolescents, and recommends reduction of sugar consumption through effective taxation on sugar-sweetened beverages [71,72]. Sugary drinks are defined by WHO as all types of beverages containing free sugars and these include carbonated or noncarbonated soft drinks, fruit/vegetable juices and drinks, liquid and powder concentrates, flavored water, energy and sports drinks, ready-to-drink tea, ready-to-drink coffee, and flavored milk drinks [71].

The inclusion of fruit juices in this definition is important for the prevention of diet-related diseases. Beverages that contain free sugars, including 100% fruit juice, have been associated with a higher risk of dental decay in children [73] and an increased risk of weight gain, overweight and obesity, and type 2 diabetes [74,75]. Fruit juices have a high concentration of fructose and other free sugars, in contrast to fruits where sugar is contained within the structure of the fruit. For this reason, the dietary guidelines of many countries no longer promote the drinking of fruit juices, which are an alternative to fruit, pleasant to the senses, and convenient to consume. E.g., in the UK in the frame of “Eat Well” program, consumers are warned to limit intake to a 150 mL glass of unsweetened fruit juice, vegetable juice, or smoothie as 1 portion of fruit and vegetables per 5 recommended daily consumption [76]. Canadian “Healthy Eating Recommendations” advise consumers to make water as your drink of choice and replace sugary drinks with water [77]. Dietary guidelines for Americans recommend reducing calories from added sugars and the amount of beverages higher in these components and encourage healthier beverage choices. The advice is that at least half of the recommended amount of fruit should come from whole fruits, and the consumed juices should be 100% juice, without added sugars [78]. Replacing SSB with plain drinking water has become central to health promotion strategies, including those led by the US federal agencies [79]. The amounts of fruit juice allowed in the USDA Food Patterns for young children, align with the recommendation from the American Academy of Paediatrics, that young children consume no more than 4–6 fluid ounces (113–170 g) of 100% fruit juice per day [78]. Among the 10 principles of healthy eating accompanying the Polish Food Pyramid for children and young people, 2 concern beverages: avoiding drinking sugary drinks and remembering to drink water for a meal and between meals, at least five to six glasses of water a day. These population groups are also recommended to consume more vegetables than fruit. In the pyramid for adults, the recommendations are to drink at least 1.5 L of water per day, to eliminate or significantly reduce the consumption of sugary drinks and flavored waters and to keep the right proportions of vegetables and fruit. In the food category “fruit and vegetables,” vegetables should make up three-fourth and fruit one-fourth of the consumption, including one portion (80 g in a minimum amount of 400 g) of fruit may be juice [10].

Free sugars defined by WHO include all monosaccharides and disaccharides added to foods and beverages by the manufacturer, cook, or consumer and sugars naturally present in honey, syrups, fruit juices, and fruit juice concentrates. Free sugars do not include the naturally occurring sources found in intact fruits and vegetables and those found in (unsweetened) milk [80]. WHO has developed two strong recommendations: to reduce intake of free sugars throughout the life and to reduce the intake of them to less than 10% of total daily energy intake. As the conditional recommendation, WHO suggests a further reduction of the intake of free sugars to below 5% of total energy intake, which will bring additional health benefits [80]. These recommendations can be easily exceeded with a single bottle of 500 mL of sugar-sweetened beverage drunk during the day [81].

It is worth noting that the changes taking place in the consumption of the subgroups of nonalcoholic beverages consumption distinguished in Poland are consistent with the recommendations. Over 10 years (2006–2016), the consumption of juices (all together) in Polish households decreased on average by 12%, but in self-employed and white-collar employee households to the greatest extent by 28% and is currently the lowest (0.72 L/person per month). At the same time, in the households of farmers (and only in this group), it has increased by almost the same amount (27%) and is now the highest (1.27 L). In the groups of blue-collar employees and pensioners, it remained at the same level, close to the current average (0.88 L). Our analysis has shown that the socioeconomic type of household is the third factor most strongly differentiating the consumption of beverages (Table 2). Our results also showed that the impact of income is the smallest (13th factor), but it is worth pointing that juice consumption in the households with the lowest income group (1st quintile group) is 2.1 times lower than in the households with the highest income group (5th quintile group) (in 2016), and 10 years ago (in 2006), it was even 4.2 times lower (in 2006) [82]. In the Polish households, the coefficient of income elasticity of demand for juice is still high [83], and for water, it is even higher [83,84].

Our research has shown that nonalcoholic beverages in the Polish diet should also be considered in the context of providing minerals, in particular calcium, magnesium, and potassium, since the share of this food category for them is 5.9%, 5.5%, and 4.5%, respectively. In the case of calcium and magnesium, the mineral and spring water subgroup has the largest share, and in the case of potassium, the subgroup of other nonalcoholic beverages. By comparison, in the average American, diet nonalcoholic beverages together with coffee and tea provided 8.4% of potassium and 4.4% of calcium [46]. In turn, in the average Australian diet, fruit and vegetable juices and drinks provided 0.9–1.0% of calcium and 1.7–2% of magnesium [58], and these data are comparable to our results (calcium—0.9%, magnesium—1.3%). The data concerning the average French diet refer to the total drinking of water, alcoholic beverages, soft drinks, and fruit juices in the category of beverages. The share of this product group in supplying potassium to the diet is 7.5% [59]. Studies show that in the case of calcium and magnesium, mineral and springs waters can provide valuable supplementation to the diet of various population groups in Poland [85,86], and to a small extent, also potassium [86]. The tap water used for meals and beverages preparation can be also an important source of calcium and magnesium in the diet, depending on their content in the water [87]. In Spain, the consumption of drinking water and natural mineral water can be also regarded as an important supplementary source of calcium [88] and magnesium [89].

The supplementary role of mineral and spring waters in the average Polish diet is increasing, as juice consumption is decreasing. In the years 2006–2016, the consumption of mineral and spring waters increased on average in households by 87%, and the growth, in particular, in socioeconomic groups depended on the initial level of the consumption. In households of farmers, it was the lowest and increased by 167% (to 3.45 L/person per month), while in households of white-collar employees and self-employed, it was the highest and increased to the lowest extent by 59% and 54%, respectively (to 5.93 and 5.14 L/person per month) [43]. The results of three NHANES surveys (2011–2016) also show that drinking tap and bottled water is replacing SSBs in the American diet, as SSBs consumption is declining significantly. The main sources of dietary water were bottled water from stores and tap water away from home and at home, in decreasing order, but total volume of tap water was almost 75% higher than bottle water. Higher tap water consumption was associated with higher incomes. Non-Hispanic whites consumed most tap water, whereas Mexican Americans consumed most bottled water [79]. These results suggest that social stratification of American population affects environmental awareness and attitudes towards acceptance of sustainable consumption patterns.

As regards to vitamin supply by nonalcoholic beverages, in the average Australian diet, fruit and vegetable juices and drinks provide 26.6–26.8% of vitamin C and 4.3–4.6% of folates [58]. In the average Polish diet, the category of nonalcoholic beverages provides much less vitamin C than in the Australian diet, accounting for only 15.9% of total supply; in the case of folates, the opposite is true—nonalcoholic beverages provide almost twice as much of this vitamin: 8.5%. In the average Polish diet, vitamin C comes in roughly equal parts from fruit juices and the subgroup of other nonalcoholic beverages (about 8–7%). Our analyses also show that among households of young employees and families with children (cluster 3), these subgroups of beverages provide vitamin C twice as much (fruit juices 15% and the subgroup of other nonalcoholic beverages 13%) as in the other clusters. This is partly due to the presence of juices and other beverages enriched with vitamin C in the market, but it must be borne in mind that consumption of these beverages will be linked to negative aspects including excessive consumption of calories in the case of sugary soft drinks and damage to tooth integrity caused by their acidity [1]. Although there is an increasing supply and number of options of lower sugar and sugar-free beverages [90], it is worth considering their environmental footprint, including packaging, when making a purchase decision for any type of beverage.

## 5. Conclusions

Drinking the right amount of fluids every day is essential for proper hydration and functioning of the body, but their ingredients contribution to the diet are equally important from a nutritional point of view. Analyses presented in this paper have shown that in the average Polish diet, nonalcoholic beverages account for 6.8% of total daily carbohydrates intake, with the subgroup of other nonalcoholic beverages contributing the largest share (5.3%). In this subgroup, similarly as in the subgroup of fruit juices, the carbohydrates were largely free sugars, the intake of which should be limited at highest extent aiming at the prevention of overweight and obesity and other noncommunicable diseases.

The fact that fruit juices provide 8% vitamin C and almost 2% vitamin B6. Folates and potassium to the Polish diet can be somewhat downplayed because less processed food and fresh fruit can be the source of these nutrients. Vegetable juices have a very small share in suppling of nutrients, providing to the diet less than 1% for each nutrient except for vitamin A (2.3%). The importance of mineral and spring waters as a source of nutrients concerns only minerals, and in the average Polish diet, these are mainly calcium (3.9%), magnesium (3.0%) as well as a small amount of sodium (1.3%) and potassium (0.1%). It can be expected that the share of water in the supply of essential microelements will increase, first, due to dietary guidelines worldwide (as well as in Poland), which encourage people to replace sweet drinks with water, with tap water being better chosen instead of bottled water (sustainable consumption), and second, data from household budget surveys in Poland show an increasing trend in mineral and spring waters consumption.

## Figures and Tables

**Table 1:** Energy and nutrients supplied by nonalcoholic beverages in the average Polish diet (in %).

	Total Nonalcoholic Beverages	Subgroups			
		Fruit Juices	Vegetable Juices and Mixed	Mineral and Spring Water	Other Nonalcoholic Beverages
**Energy**	3.31	0.64	0.13	0.00	2.54
Carbohydrates	6.80	1.27	0.27	0.00	5.26
Calcium	5.93	0.71	0.21	3.86	1.15
Phosphorus	1.03	0.31	0.09	0.00	0.63
Sodium	1.58	0.02	0.15	1.29	0.13
Potassium	4.45	1.76	0.46	0.09	2.14
Iron	2.59	0.87	0.26	0.10	1.35
Magnesium	5.53	1.04	0.21	3.03	1.25
Manganese	2.55	1.02	0.24	0.00	1.30
Iodine	1.27	0.13	0.12	0.01	1.02
Copper	3.47	1.23	0.33	0.00	1.91
Zinc	1.03	0.34	0.14	0.00	0.55
Vitamin A	3.70	0.70	2.31	0.00	0.68
Vitamin E	2.05	0.68	0.55	0.00	0.83
Thiamin	2.21	1.10	0.19	0.00	0.93
Riboflavin	1.32	0.61	0.14	0.00	0.58
Niacin	1.39	0.61	0.18	0.00	0.59
Vitamin B6	8.85	1.80	0.30	0.00	6.75
Folate	8.52	1.68	0.37	0.00	6.47
Vitamin B12	1.99	0.16	0.00	0.00	1.83
Vitamin C	15.85	8.08	0.95	0.00	6.82

**Table 2:** Dependence of cluster analysis on sociodemographic and economic factors of the households.

Factors	Cramer Correlations
Family life phase	**0.235**
Age	**0.230**
Socioeconomic group	**0.189**
Number of people in household	**0.171**
Sex	0.093
Education level	0.080
Self-assessment of financial situation	0.079
Self-assessment of nutrition in household	0.074
Usage of agricultural land	0.070
Region	0.063
Month of participation in the survey	0.057
Size of the village	0.038
Degree of urbanization of place of household living	0.034
Income (quintile group)	0.030

The most important factors are written in bold.

**Table 3:** Cluster description.

	Cluster 1	Cluster 2	Cluster 3	Whole Population
Number of households in clusters	18908	7031	10946	36886
Number of people (in %)	51.3	19.0	29.7	100.0
**Family Life Phase** **(in %)**				
Singles and young marriages	11.8	14.3	17.2	13.9
Families with preschool children	8.5	18.6	25.8	15.6
Families with school children	9.6	15.8	21.6	14.3
Families with trainees	15.9	15.9	12.7	15.0
Older people and older marriages (blue-collar workers, white-collar workers, or self-employed)	25.9	18.6	12.2	20.4
Older people and older marriages and Retired	28.3	16.8	10.5	20.8
**Age (in %)**				
Less than 30 years	4.8	8.5	11.8	7.6
30 – >40 years	10.9	19.6	27.7	17.5
40 – >50 years	13.3	19.7	24.3	17.8
50 – >60 years	21.2	20.4	16.0	19.5
60 – >70 years	27.3	18.8	12.7	21.3
70 years and older	22.5	13.1	7.5	16.2
**Socioeconomic Group (in %)**				
Blue-collar workers	19.6	27.2	31.2	24.5
White-collar workers	18.6	26.2	31.9	24.0
Farmers	5.1	4.5	3.8	4.6
Self-employed	5.5	7.4	8.7	6.8
Pensioners	39.6	25.2	16.0	29.9
Disability pensioners	8.0	5.4	3.9	6.3
Living on social benefits	2.4	2.7	2.8	2.6
Living from other unearned sources	1.3	1.6	1.7	1.5
**Number of People in Household (in %)**				
1	25.7	16.6	14.2	20.6
2	38.7	30.3	24.2	32.8
3	16.6	22.6	23.5	19.8
4	11.4	19.0	24.2	16.6
5 and more	7.6	11.6	13.9	10.3
Main groups in clusters	Cluster 1 Elders Without Children Older people or marriages; 60 years and older; working or pensioners; 1–2 people	Cluster 2 Workers in small families Families with preschool children or pensioners; 30–60 years blue-collar and white-collar workers; 2–3 people	Cluster 3 Aspiring young with children Families with children; 30–50 years blue-collar and white-collar workers; 2 or 4 people	
